# Printable 3D vocal tract shapes from MRI data and their acoustic and aerodynamic properties

**DOI:** 10.1038/s41597-020-00597-w

**Published:** 2020-08-05

**Authors:** Peter Birkholz, Steffen Kürbis, Simon Stone, Patrick Häsner, Rémi Blandin, Mario Fleischer

**Affiliations:** 1 Institute of Acoustics and Speech Communication, TU Dresden, Dresden, Germany; 2 Charité – Universitätsmedizin Berlin, Department of Audiology and Phoniatrics, Berlin, Germany

**Keywords:** Communication, Fluid dynamics, Biomedical engineering

## Abstract

A detailed understanding of how the acoustic patterns of speech sounds are generated by the complex 3D shapes of the vocal tract is a major goal in speech research. The Dresden Vocal Tract Dataset (DVTD) presented here contains geometric and (aero)acoustic data of the vocal tract of 22 German speech sounds (16 vowels, 5 fricatives, 1 lateral), each from one male and one female speaker. The data include the 3D Magnetic Resonance Imaging data of the vocal tracts, the corresponding 3D-printable and finite-element models, and their simulated and measured acoustic and aerodynamic properties. The dataset was evaluated in terms of the plausibility and the similarity of the resonance frequencies determined by the acoustic simulations and measurements, and in terms of the human identification rate of the vowels and fricatives synthesized by the artificially excited 3D-printed vocal tract models. According to both the acoustic and perceptual metrics, most models are accurate representations of the intended speech sounds and can be readily used for research and education.

## Abstract (summary)

**Table:** 

Measurement(s)	Volume velocity transfer functions of the vocal tract • 3D shape of the vocal tract • Noise • Larynx
Technology Type(s)	external acoustic excitation using sine sweeps • magnetic resonance imaging (MRI) • airflow injection through the glottis of 3D-printed vocal tract models
Factor Type(s)	fluid power level • type of speech sound
Sample Characteristic - Organism	Homo sapiens

Machine-accessible metadata file describing the reported data: 10.6084/m9.figshare.12591554

## Background & Summary

Recently, Magnetic Resonance Imaging (MRI) has become an important tool for speech research. It can be used to acquire detailed 3D images of the entire vocal tract of static articulations, which the speaker has to sustain for multiple seconds (3D MRI)^1^, or alternatively, to acquire the dynamic articulation from the entire mid-sagittal plane of the vocal tract (real-time MRI, with typically more than 30 frames per second^2^) or individual organs like the vocal folds^3^. Among all available speech articulation measurement techniques, 3D MRI allows one to obtain the most detailed and complete reconstruction of the 3D geometry of the vocal tract (apart from Computed Tomography scans, which use potentially harmful radiation), allowing to address a range of new questions in speech research. Accordingly, since about the 1990s, many studies have collected and analyzed 3D MRI data of the vocal tract for different purposes^4–11^.

However, it is both time-consuming and costly to acquire and process 3D MRI data of the vocal tract, so that public MRI speech datasets are highly desirable. In recent years, the following public datasets including 3D MRI data of the vocal tract have been published:

- The ATR MRI database with 3D vocal tract images of the five Japanese vowels from one subject^12^.
- The mngu0 corpus with 3D vocal tract images of 28 speech sounds from one English subject^13^.
- The USC Speech and Vocal Tract Morphology MRI Database with 3D vocal tract images of 27 speech sounds, each from 17 English speakers^1^.
- The MRI articulatory corpus of French with 3D vocal tract images of 44 (context-dependent) speech sounds, each from two French speakers^14^.
- The MRI dataset of the Aalto University, Finland, with 3D vocal tract images of 8 Finish vowels, each from two subjects^15,16^ (http://speech.math.aalto.fi/data.html). This dataset also contains triangle meshes of the inner vocal tract surfaces extracted from the MRI data as STL files.

**Fig. 1:**
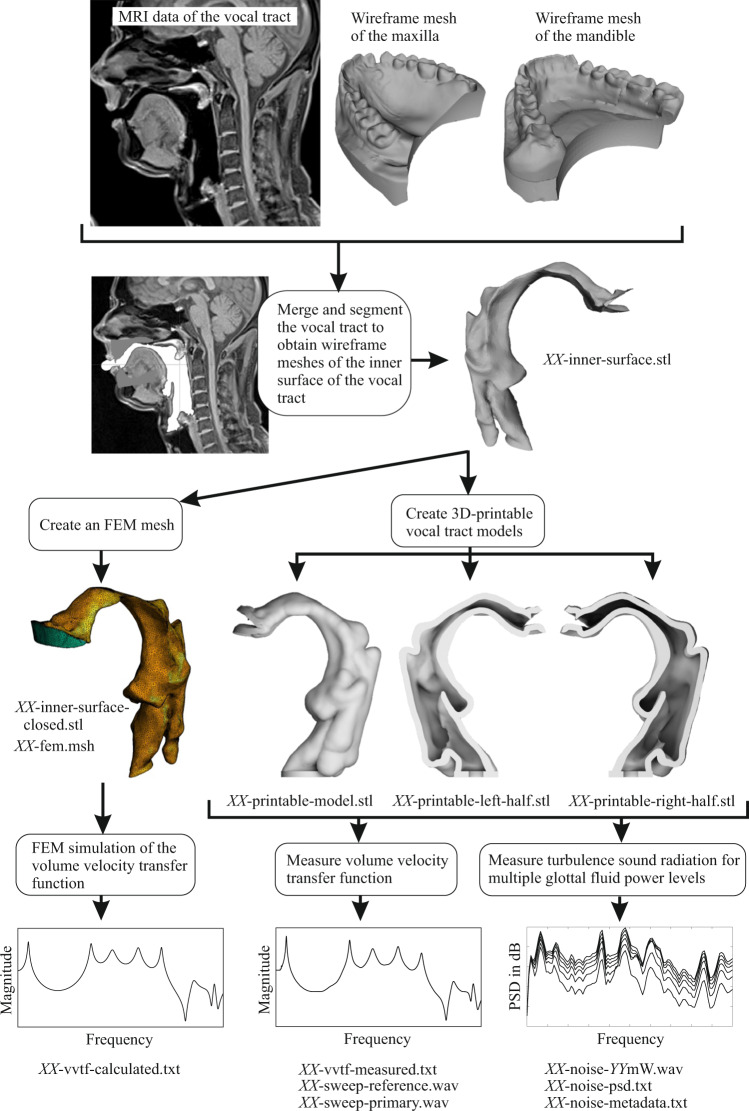
Overview of the data acquisition and processing. The shown images and spectra are for the tense vowel/e:/of speaker 1. *XX* in the file names is a placeholder for the speech sound labels.

Here, we present a dataset containing 3D vocal tract images of 22 German speech sounds (16 vowels and 6 consonants), each from one male and one female speaker. In contrast to the datasets mentioned above, which contain only the *raw* MRI data (except the Finish dataset), the data here were extensively processed and evaluated to make them accessible to non-experts on volumetric MRI processing. The processing steps are summarized in Fig. 1. Because teeth are not visible in MRI data, but highly relevant for speech acoustics^17^, we created 3D scans of plaster models of the teeth of the subjects. The teeth scans were merged with the MRI data, and the vocal tract was segmented to yield triangle meshes of the vocal tract walls. From the segmented vocal tract surfaces, we created finite element (FE) models for acoustic simulations, as well as 3D-printable solid volume models. The FE models were used to *calculate* the volume velocity transfer functions of the vocal tract from the glottis to the lips (up to 10,000 Hz). The solid volume models were 3D-printed and used to *measure* the acoustic transfer functions. The calculated and measured transfer functions were compared in terms of their resonance frequencies. To characterize the models aeroacoustically, stationary airflows at different fluid power levels were injected through the glottis of the 3D-printed models, and the noise generated by the turbulence was measured in front of the lips of the models. Finally, to check whether the processed vocal tract shapes in the dataset are still sufficient representations of the respective speech sounds, the 3D-printed vocal tract models were acoustically excited (vowels with a reed source at the glottis and fricatives with a stationary airflow through the glottis) to generate artificial speech sounds, and human listeners were asked to identify them in a perception experiment.

There is a range of potential applications for the provided data. For example, they could be used

- to create articulatory models of the vocal tract or individual articulators based on the provided vocal tract shapes^18–24^.
- to validate computational acoustic models of the vocal tract, especially simplified (and fast) 1D and 2D models^11,25–27^, using the provided vocal tract geometries along with the transfer functions as reference.
- to validate computational aeroacoustic models that simulate noise generation in the vocal tract^28,29^, using the provided vocal tract geometries along with the turbulence noise measurements as reference.
- to assess the acoustic effects of certain geometric features or side cavities of the vocal tract, like the piriform fossae^6,30^, the interdental spaces, or the vallecula, based on the 3D-printable models.
- as teaching tools to demonstrate the relationship between vocal tract shape and acoustics, based on the 3D-printable models. With suitable physical mechanisms for the voiced excitation^31^, e.g., a reed pipe as described by Arai^32,33^, the 3D-printed models can be used to synthesize different vowels.
- in combination with other MRI or CT datasets, to study questions of morphology and anatomic development, gender differences, or inter-speaker anatomic or articulatory variability of the vocal tract^34–38^.

## Methods

This study was conducted according to the ethical principles based on the WMA Declaration of Helsinki and to the current legal provisions. It was approved by the ethics committee of the TU Dresden, and informed consent was obtained from the subjects.

### Subjects and speech sounds

Vocal tract shapes of sustained speech sounds were acquired from two native German speakers, one male and one female. The male subject (s1) was 39 years old, 1.85 m tall, and grew up in the state Mecklenburg-Vorpommern (Mecklenburg-Western Pomerania) in Germany. He was a professional phonetician and speech scientist and lecturer at the university.

The female subject (s2) was 32 years old, 1.64 m tall, and grew up in the state Sachsen (Saxony) of Germany. She did her studies in speech science, which included professional speech training. Furthermore, she is a trained singer and has been singing in several semi-professional choirs since her childhood.

Each subject produced 22 sustained speech sounds while a volumetric MRI scan of their vocal tract was performed. The data were processed and analyzed as discussed below. The list of speech sounds is given in Table 1 and contains 8 tense vowels, 8 lax vowels, and 6 consonants. The subjects were asked to pronounce each sound like in the word given in the table. The two rightmost columns contain the unique labels for the vocal tract shapes used in the dataset and in the remainder of this paper. In the following, they will be referred to by the placeholder *XX*.

**Table 1:** List of vocal tract shapes (sustained phonemes) and their labels (*XX*) used in the file names and folder names.

Phoneme	as in		Label for subject 1	Label for subject 2
aː	Bahn	/baːn/	s1-01-bahn-tense-a	s2-01-bahn-tense-a
eː	Beet	/beːt/	s1-02-beet-tense-e	s2-02-beet-tense-e
iː	Tiere	/tiːʁə/	s1-03-tiere-tense-i	s2-03-tiere-tense-i
oː	Boote	/boːtə/	s1-04-boote-tense-o	s2-04-boote-tense-o
uː	Bude	/buːde/	s1-05-bude-tense-u	s2-05-bude-tense-u
εː	Lähmung	/lεːmʊŋ/	s1-06-laehmung-tense-ae	s2-06-laehmung-tense-ae
øː	Höhle	/høːlə/	s1-07-hoehle-tense-oe	s2-07-hoehle-tense-oe
yː	Güte	/gyːtə/	s1-08-guete-tense-y	s2-08-guete-tense-y
l	los	/loːs/	s1-09-los-l	s2-09-los-l
f	Fahrt	/faːʁt/	s1-10-fahrt-f	s2-10-fahrt-f
s	Bass	/bas/	s1-11-bass-s	s2-11-bass-s
∫	schön	/∫øːn/	s1-12-schoen-sh	s2-12-schoen-sh
ç	ich	/Iç/	s1-13-ich-c	s2-13-ich-c
x	ach	/ax/	s1-14-ach-x	s2-14-ach-x
a	Bass	/bas/	s1-15-bass-lax-a	s2-15-bass-lax-a
ε	Bett	/bεt/	s1-16-bett-lax-ae	s2-16-bett-lax-ae
I	mit	/mIt/	s1-17-mit-lax-i	s2-17-mit-lax-i
ɔ	offen	/ɔfn/	s1-18-offen-lax-o	s2-18-offen-lax-o
ʊ	Butter	/bʊtɐ/	s1-19-butter-lax-u	s2-19-butter-lax-u
Y	Mütter	/mYtɐ/	s1-20-muetter-lax-y	s2-20-muetter-lax-y
œ	Götter	/gœtɐ/	s1-21-goetter-lax-oe	s2-21-goetter-lax-oe
ə	Ehe	/eːə/	s1-22-ehe-schwa	s2-22-ehe-schwa

### Acquisition of MRI and reference audio data

The MR images of the vocal tract were acquired on a Siemens 3 T TIM Trio with a 12-channel head coil combined with additional neck elements. The sequence was a sagittal 3D volume interpolated gradient echo sequence (VIBE - fl3d-vibe) with 1.2 mm × 1.2 mm × 1.8 mm resolution, 44 sequential slices, matrix size 192 × 192, field of view = 230 mm × 230 mm, repetition time TR = 5:53 ms, echo time TE = 2:01 ms, flip angle 9°, Q-fatsat, 22 lines per shot, 7/8 phase partial Fourier, 6/8 slice partial Fourier, ipat factor 2 (PE only), 24 reference lines and a bandwidth of 220 Hz/pixel. The acquisition time for one volume was 14 s during which the speaker produced and sustained the corresponding speech sound. All 22 sounds per speaker were acquired in one session. After each scan, the image quality was carefully checked with respect to blurry parts or motion artifacts due to involuntary movements of the articulators during the 14 s scan time. Each scan was repeated as often as necessary to obtain a clean image.

Before the MRI sessions, the two subjects practiced to sustain the speech sounds for the required duration and with a High German quality. It was especially practiced to produce the lax vowels with the correct vowel quality, as they are normally produced as short vowels in German.

In addition to the MRI data, audio recordings of the speech sounds were obtained from both subjects. These recordings were not directly made during the MRI scans of the vocal tract because of the high noise level in the scanner. Instead, they were done in a separate session in a soundproofed audio studio using a studio condenser microphone (M930 by Microtech Gefell) connected to a mixing desk (Behringer Eurorack MX 1602) for power supply and preamplification. The signals were digitized with 44,100 Hz and 16 bit using the audio interface 896HD by MOTU and recorded with the Software Audacity 2.2.0 (https://audacityteam.org/) on a standard desktop computer.

The subjects were asked to produce the sounds as similar as possible to the situation in the MRI scanner and sustain the sounds for at least 10 s. The recordings were then symmetrically cropped around their center to a length of 1,000 ms for the tense vowels (which normally occur as long vowels in German) and the fricatives, and to a length of 200 ms for the lax vowels. They were then peak-normalized and windowed with a Tukey (tapered cosine) window for a fade-in and fade-out of 20 ms each. Finally, the audio signals were padded with 200 ms of silence at the beginning and end. The resulting audio signals are contained in the dataset as the files *XX*-reference-sound.wav.

### Measurement of maxilla and mandible shapes

For each subject, plaster models of the maxilla and mandible were created by means of alginate impressions according to the standard procedure used in dentistry^39^. The plaster models were 3D-scanned to obtain 3D boundary models of the objects (see top row of Fig. 1). Scanning was performed with a NextEngine 3D laser scanner and the corresponding NextEngine ScanStudio software. Each plaster model was scanned both from a horizontal (as in Fig. 1) and a vertical view (standing on the posterior side). In each position, the model was scanned from 7 angles in steps of 51.4° on the turntable with the following settings: Points per Square Inch: 4400 (SD); Target: Normal; Range: Macro. The individual scans per object were then aligned using corresponding points on the surface, fused into a single boundary model, and exported as a binary STL file. These are contained as the files s1-mandible.stl, s1-maxilla.stl, s2-mandible.stl, and s2-maxilla.stl in the dataset.

### Segmentation of the vocal tract

To obtain the inner surface representations of the vocal tracts from the MRI data, each vocal tract was processed according to the steps below. All required software tools were free and open source.

1. The boundary models of the maxilla and mandible were merged with the MRI data of the vocal tract shape using the software 3D Slicer^40^ (www.slicer.org). The MRI voxel data were first upsampled to obtain smaller voxels with a uniform edge length of 0.25 mm. Then the triangle meshes of the maxilla and mandible were carefully positioned with respect to the MRI data using affine transforms. Finally, all voxels contained within the closed surfaces of the maxilla and mandible were set to a constant mid-level gray value.
2. The high-resolution voxel data from step 1 were used to segment the vocal tract with the software ITK-SNAP^41^ (http://www.itksnap.org). The 3D segmentation was performed semi-automatically based on the implemented active contour method^42^. The nasal cavity was excluded from the segmentation, even when there was a slight velo-pharyngeal opening for some vowels. The segmentation result was a boundary model of the air-filled pharyngeal and oral cavities that extended slightly into the free space in front of the open mouth.
3. The closed boundary model obtained in step 2 was opened at the glottal end and the mouth using the software Blender (www.blender.org). The glottal end was opened with a cutting plane through the vocal folds, while the mouth was opened with curved cutting planes that were fitted to the shape of the lips.
4. The surface model opened at the glottis and the mouth was manually smoothed with a sculpting tool using Blender and a Laplacian filter using the software Meshlab^43^ (http://www.meshlab.net). It was taken care that important details like the teeth, the uvula, and the epiglottis were not accidentally removed.

The triangle meshes of the inner vocal tract surfaces are provided as the files *XX*-inner-surface.stl in the dataset.

### Creation of 3D-printable models and 3D-printing

To obtain 3D-printable models of the vocal tract, the inner surface meshes were converted into closed solids by giving the vocal tract walls a finite thickness. For each model, we first created an offset mesh as the exterior shell for the solid using the software Meshlab. The offset mesh was created at a distance of 4 mm outwards from the inner surface mesh for a wall thickness of 4 mm, and then trimmed using Blender. The outer shell was then smoothed and fused with the inner shell using Blender. The gaps between the meshes were closed and a uniform adapter (socket) was added to the glottal end of the model. The adapter was designed as a disk-shaped ring with a thickness of 4 mm and inner and outer diameters of 10 mm and 30 mm, respectively. The upper side of the ring was positioned flush with the glottal plane (inlet of the vocal tract). Hence, the glottal opening of all models consisted of a hole with 10 mm diameter. The complete set of volume models including the adapter is supplied as the files *XX*-printable-model.stl in the dataset. For easier 3D-printing, the models have also been halved through the midsagittal plane, and the two halves are represented by the files *XX*-printable-left-half.stl and *XX*-printable-right-half.stl.

Each vocal tract half was 3D-printed on an Ultimaker 3 printer, which uses fused deposition modeling and has two extruders. The vocal tract walls were printed with the material PLA (polylactic acid, brand “innofil”) from one extruder, and support structures were printed with the water-soluble material PVA (polyvinyl alcohol, using the material sold by Ultimaker) from the other extruder. The layer thickness was 0.1 mm and the infill ratio was 100% for PLA (i.e. the walls were “solid” inside) and 20% for PVA. Both extruders had a nozzle diameter of 0.4 mm. The vocal tract halves were oriented with the cutting plane, i.e. the midsagittal plane on the build plate. The build plate was heated to 60° C with a heating bed for better adhesion. The print time was about 20 h per half. The material consumption was about 50 g PLA and 10 g PVA per half, i.e. the mass of a complete vocal tract model was about 100 g. After printing all objects and dissolving their support structures, the two halves of each vocal tract model were carefully sanded at the side that adhered to the build plate and glued together with cyanoacrylate adhesive (“superglue”).

Due to the PLA material used for 3D printing, the walls of the vocal tract models were essentially hard compared to the soft walls of a human vocal tract. For the sake of reproducibility, we made no attempt here to create more realistic soft walls, because suitable methods to achieve this for detailed vocal tract geometries have not been explored yet. However, future studies could readily use the models in the dataset to create and examine soft-walled models.

### Measurement of the volume velocity transfer functions

For each of the 44 physical vocal tract models, the volume velocity transfer function (VVTF) was measured. The VVTF $H(\omega )$ is often used to characterize vocal tract acoustics^12,44,45^ and usually defined as the complex ratio of the volume velocity ${U}_{2}(\omega )$ through the lips to the volume velocity ${U}_{1}(\omega )$ through the glottis, i.e.,

1 $$H(\omega )={U}_{2}(\omega )/{U}_{1}(\omega ).$$

here, the transfer functions were determined for the case of an infinite glottal impedance, i.e., a closed glottal end of the tubes. The determination of the VVTF based on Eq. (1) is technically very challenging^46^, because it would require a broadband volume velocity source ${U}_{1}(\omega )$ at the glottis and a broadband volume or particle velocity sensor at the mouth. A simpler yet precise approach to determine $H(\omega )$ was presented by Fleischer *et al*.^47^, which was also adopted in the present study. Fleischers’ method does not require a volume velocity source or sensor, but can determine $H(\omega )$ solely from two sound pressure measurements ${P}_{1}(\omega )$ and ${P}_{2}(\omega )$ at the glottis and the lips, respectively, as described below. This method is based on the principle of reciprocity and theoretically well-founded^47^.

**Fig. 2:**
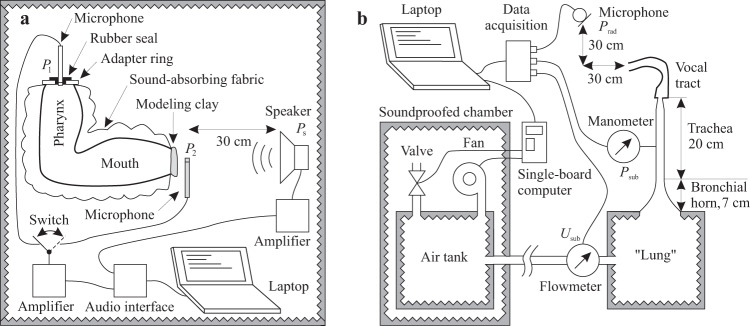
(**a**) Setup for measuring the (closed-glottis) volume velocity transfer function between the glottis and the lips. First, the reference sound pressure in front of the closed mouth was measured as ${P}_{2}(\omega )$. Then the clay and the mouth microphone were removed (gray items) and the sound pressure ${P}_{1}(\omega )$ was measured inside the vocal tract at the level of the glottis. (**b**) Setup for aeroacoustic measurements.

The experimental setup for the measurements is shown in Fig. 2a. The vocal tract model was placed at a fixed distance of about 30 cm in front of a loudspeaker. A 1/4-inch measurement microphone (MK301E capsule with MV301 preamplifier by Microtech Gefell) was inserted into the hole at the glottal end of the model so that its membrane was flush with the glottal plane. A measurement consisted of two steps. In the first step, the loudspeaker emitted a broadband excitation signal (sine sweep) into the *open* mouth of the model while the sweep response ${P}_{1}(\omega )$ was measured with the glottis microphone. In the second step, the mouth of the model was tightly closed with a plug made of modeling clay (about 1 cm thick) and another 1/4-inch measurement microphone (G.R.A.S. 46BL) was centrally positioned about 2 mm in front of the closed mouth. This microphone recorded the response ${P}_{2}(\omega )$ for the same excitation signal as in step 1. The VVTF was finally calculated as $H(\omega )={P}_{1}(\omega )/{P}_{2}(\omega )$ (which is the same as ${U}_{2}(\omega )/{U}_{1}(\omega )$). Both microphones were connected to an audio interface (Terratec Aureon XFire 8.0 HD), which in turn was connected to a laptop computer (MSI GT72-2QE) with the operating system Windows 8.1, 64 Bit.

The measurements were performed with the open-source software MeasureTransferFunction^48^, which implements the method by Farina^49^. The excitation signal used in this software was a logarithmic sine sweep with a power band from 100 Hz to 10,000 Hz (fade-in and fade-out from 50–100 Hz and 10,000–11,000 Hz, respectively) and a duration of 10.4 s. The source signal amplitude was set to 0.5, i.e. to 50% of the value range. The output level and input level of the audio interface were set to 100% and 50%, respectively. The audio signals were sampled with 96,000 Hz and quantized with 24 bit. A major benefit of using logarithmic sweeps to characterize acoustic systems is that the linear impulse response can be separated from signal components generated by harmonic distortions^49^. Accordingly, the linear response was manually extracted in all recorded signals before further processing. The different sensitivities of the microphones used at the glottis (3.2 mV/Pa) and the mouth (18 mV/Pa) were compensated by adding 15 dB to the calculated VVTF.

Due to small variations of the latency of the audio system, there was usually a small time lag $\tau$ between the sweep responses, from which ${P}_{1}(\omega )$ and ${P}_{2}(\omega )$ were calculated. According to the time-shift property of the Fourier Transform, the shift of a time signal by $\tau$ causes its spectrum to be changed by the factor ${e}^{j\omega \tau }$, where $j=\sqrt{-1}$. This means that a phase response $\varphi (\omega )=\text{arg}H(\omega )$ is the sum of the “true” phase response and a linear function $\omega \tau$, where the slope $\tau$ may vary across models. Therefore, to explore the phase responses of the models, it might be more convenient to do it on the basis of the group delay $-d\varphi (\omega )/d\omega$, where the linear function translates into a constant offset.

All measurements were performed in the large climate-controlled anechoic chamber at the TU Dresden at a temperature of 22 °C, an atmospheric pressure of 1007 hPa, and an air humidity of 46%. The anechoic chamber is a free-field room (all six sides covered with 1 m absorbing foam spikes) with a free volume of 1000 m^3^ and a degree of sound absorption of at least 99% for frequencies between 60 Hz and 16 kHz. Before the measurements, the vocal tract models were tightly wrapped in multiple layers of sound-absorbing fabric. This minimized the external excitation of the (plastic) vocal tract walls by the source signal during the measurement of *P*_1_. Wall vibrations due to the external excitation would otherwise interfere with the sound field in the models and create spectral artifacts. The two sweep responses *P*_1_ and *P*_2_ are contained in the files *XX*-sweep-primary.wav and *XX*-sweep-reference.wav in the dataset. The transfer functions $H(\omega )$ are given in the files *XX*-vvtf-measured.txt.

### Calculation of the volume velocity transfer functions

For comparison with the measurements of the physical models, the VVTFs were also determined numerically using the finite element method (FEM). The calculation was similar to that described by Fleischer *et al*.^47^ on the basis of the freely available software FEniCS^50^ (http://fenicsproject.org). To create the FE models, the inner surface meshes of the vocal tract (*XX*-inner-surface.stl) were first “closed” at the glottal end and the mouth, as in the files *XX*-inner-surface-closed.stl. These closed-surface meshes were then converted into volume meshes (*XX*-fem.msh) for the FE simulations with the free software Gmsh^51^ (http://gmsh.info/). In the volume meshes, the regions of the glottis, the mouth opening, and the vocal tract walls were manually marked to define the boundary conditions for the acoustic simulation.

The FE models were discretized with linear shape functions and had a number of degrees of freedom between 99,688 (model s1-22-ehe-schwa) and 147,806 (model s2-08-guete-tense-y). Furthermore, the maximum mean element size was 2.99 mm (model s1-22-ehe-schwa). For a maximum analysis frequency of 10,000 Hz and a sound velocity of 345 m/s at 22 °C, there were on average 11 elements/wavelength.

The acoustic simulation was based on the numerical analysis of the Helmholtz equation

2 $$-({\kappa }^{2}+{\nabla }^{2})P(\overrightarrow{x},\omega )=0,$$

where *P* is the complex-valued scalar acoustic pressure, $\overrightarrow{x}$ is the position in ${{\mathbb{R}}}^{3}$, $\omega$ is the angular frequency, $\kappa =\omega /c$ is the wave number, and *c* = 345 m/s is the speed of sound at 22 °C. The application of a frequency-independent particle velocity *V*_0_ at the glottis leads to the boundary condition

$$\nabla {P}_{{\rm{glottis}}}\cdot \overrightarrow{n}=-\,j\omega \varrho {V}_{0}$$

at the glottal surface, where $\varrho =1.18$ kg/m^3^ is the density of air. At the vocal tract walls, the boundary condition

$$\nabla {P}_{{\rm{wall}}}\cdot \overrightarrow{n}=-\,j\kappa \frac{\varrho c}{{Z}_{{\rm{wall}}}}{P}_{{\rm{wall}}}$$

with the empirical value ${Z}_{{\rm{wall}}}=500\cdot \varrho c$ was applied^47^. At the lip opening, the boundary condition

$$\nabla {P}_{{\rm{lips}}}\cdot \overrightarrow{n}=-\,j\kappa \frac{\varrho c}{{Z}_{{\rm{r}}}}{P}_{{\rm{lips}}}$$

was implemented. The radiation impedance was assumed to be

$${Z}_{r}=\varrho c\left(\frac{{(\kappa r)}^{2}}{1+{(\kappa r)}^{2}}+j\frac{\kappa r}{1+(\kappa r)}\right),$$

where $r=\sqrt{{A}_{{\rm{lips}}}/(2\pi )}$ and ${A}_{{\rm{lips}}}$ represents the lip opening area^52,53^. Based on the computed pressure ${P}_{{\rm{lips}}}$ at the central point of the lip opening, the default value ${V}_{0}$, and the geometrical measures ${A}_{{\rm{lips}}}$ and ${A}_{{\rm{glottis}}}$, the transfer function

$${H}_{{\rm{FEM}}}(\omega )={A}_{{\rm{lips}}}\cdot \frac{{P}_{{\rm{lips}}}(\omega )}{{Z}_{{\rm{lips}}}(\omega )}/({A}_{{\rm{glottis}}}\cdot {V}_{0})$$

was calculated for frequencies between 0 and 10,000 Hz in steps of 0.961304 Hz. The computing time per model was up to 9 hours using 12 cores of the Intel Skylake Gold 6148 CPU available at the North-German Supercomputing Alliance (HLRN). The results are contained in the files *XX*-vvtf-calculated.txt in the dataset.

### Measurement of flow-induced noise for different fluid power levels

To characterize the 44 vocal tract models in aeroacoustic terms, the setup in Fig. 2b was used to create different levels of stationary airflow through the models. For each level, we recorded the volume velocity ${U}_{{\rm{sub}}}$, the subglottal pressure ${P}_{{\rm{sub}}}$, and the turbulence sound ${P}_{{\rm{rad}}}$ radiated from the mouths of the models. The airflow was generated by a fan (type U71HL-024KM-43 by Micronel) and led into an air tank, which was connected to a “lung” via a 200 cm long rubber tube with an inner diameter of 19 mm. The air tank and the lung were boxes with inner volumes of 30 × 30 × 50 cm^3^ and 23 ×23 × 23 cm^3^, respectively. Both boxes were lined with sound absorbing foam (NOMA ACOUSTIC 25 mm by NMC) and meant to attenuate the noise from the fan. A short horn connected to a straight tube (18 mm inner diameter) was used to represent the bronchia and the trachea and led the airflow from the lung to the glottal end of the vocal tract models. The dimensions of the horn and the tube were chosen to approximate the cross-sectional area function of the human subglottal system^54^. Both the horn and the tube were 3D-printed with the material PLA and with a wall thickness of 3 mm (100% infill ratio). The upper 3 cm of the tracheal tube (corresponding to the conus elasticus) tapered from 18 mm diameter to 10 mm diameter to match the diameter of the glottal hole of the attached vocal tract model. The 3D-printable volume models for these parts are contained in the files trachea.stl and bronchial_horn.stl in the dataset.

A data acquisition device (DT9837C by Data Translation) connected to a laptop computer (MSI GT72-2QE running MS Windows 8.1) was used to simultaneously measure

- the radiated sound pressure ${P}_{{\rm{rad}}}$ using a measurement microphone (1/2 inch capsule MK 250 with preamplifier MV 210 by Microtech Gefell GmbH) positioned 30 cm in front and 30 cm sideways from and directed towards the mouth of the vocal tract model (to prevent the airstream from directly hitting the microphone membrane),
- the subglottal pressure ${P}_{{\rm{sub}}}$ using a pressure measuring device (DMU4 by Kalinsky Sensor Elektronik, Erfurt, Germany) attached to a pressure tap 12 cm below the glottis,
- and the volume velocity ${U}_{{\rm{sub}}}$ at the entrance of the lung using a flowmeter (type AWM720-P1 by Honeywell).

All three signals were digitized with a sampling rate of 48,000 Hz and quantized with 24 bits. A custom-made software was used to record and display the signals, and to control the fan power. The fan power could only be adjusted in (small) steps. For a more precise adjustment of the subglottal pressure and the flow, we used a servo valve attached to the air tank. A single-board computer (type Raspberry Pi 3 Model B+) with a custom-made Python script was used to translate the high-level commands of the software on the laptop computer into electrical control voltages for the fan and the valve. The air tank with the fan and the valve were located in a separate soundproofed chamber to prevent their noise from disturbing the measurements.

For a consistent aeroacoustic characterization of the vocal tract models, we decided to apply the same six levels of fluid power (which is the product of the subglottal pressure and the volume velocity) to each model, namely 500 mW, 1000 mW, 1500 mW, 2000 mW, 2500 mW, and 3000 mW. Using fixed power levels instead of fixed levels of subglottal pressure or flow allowed to cope with the wide range of flow resistances across the models. According to the analysis by Stevens^45^, a fluid power level of 500 mW is roughly typical for “normal” speech production, while about 3000 mW is the maximum that humans can achieve.

For each power level and model, the three signals described above (radiated sound, flow, subglottal pressure) were captured for a duration of 10 s. The audio files of the radiated sounds are included in the dataset as the files *XX*-noise-500mW.wav, …, *XX*-noise-3000mW.wav. The samples in these files are floating point values proportional to the sound pressure measured at the microphone, where the value 1.0 corresponds to a sound pressure of 12.62 Pa. For each of these audio files, the power spectral density (PSD) has been estimated using Welch’s method as implemented in the function pwelch() in the Signal Processing Toolbox of Matlab R2017b. We used overlapping Hamming windows of 1024 samples (which was also the FFT length) and a window overlap of 512 samples so that the spectral resolution was 46.9 Hz. The resulting PSDs (with the unit Pa^2^/Hz) for the six power levels were summarized in the files *XX*-noise-psd.txt. Finally, the average volume velocity, the average subglottal pressure, and the overall sound pressure level (SPL) of the radiated sound for each power level were tabulated in the files *XX*-noise-metadata.txt. The SPLs were calculated from the audio signal *x*(*k*) as

3 $$SPL=20\cdot {\log }_{10}\left(\frac{1}{{P}_{{\rm{r}}{\rm{e}}{\rm{f}}}}\sqrt{\frac{1}{N}\mathop{\sum }\limits_{k=0}^{N-1}{x}^{2}(k)}\right),$$

where *k* is the sample index, *x*(*k*) has the unit Pa, $N=48000\cdot 10$ is the number of audio samples for 10 s, and ${P}_{{\rm{ref}}}=2\cdot 1{0}^{-5}$ Pa.

### Synthesis of speech sounds with the 3D-printed models

For each 3D-printed vocal tract model, the corresponding speech sound was (physically) synthesized. The tense and the lax vowels were synthesized with the setup in Fig. 2b, but with a vibrating reed source inserted between the upper end of the trachea and the glottal hole of the vocal tract models as in Birkholz *et al*.^31^. The vibrating reed source was developed by Arai^33^ and is an improved version of the design published previously^32^. The subglottal pressure was individually adjusted for each vowel as roughly the midpoint between the onset and the offset pressures of the source in combination with the respective supraglottal model. The generated sound was captured with a measurement microphone (1/2 inch capsule MK 250 with preamplifier MV 210 by Microtech Gefell GmbH) positioned 30 cm in front and 30 cm sideways from and directed towards the mouth of the vocal tract model. The sound generated by each model was recorded for 10 s. The recordings were symmetrically cropped around their center to a length of 1000 ms for the tense vowels and to a length of 200 ms for the lax vowels. They were then peak-normalized and windowed with a Tukey (tapered cosine) window for a fade-in and fade-out of 20 ms each. Finally, the stimuli were padded with 200 ms of silence at the beginning and end.

The voiceless fricatives were synthesized with the setup in Fig. 2b with a constant subglottal pressure of 800 Pa. Each of these sounds was also recorded for 10 s, cropped to 1000 ms around the center of the recording, and otherwise processed like the vowel recordings. The resulting audio signals are contained in the dataset as the files *XX*-model-sound.wav.

## Data Records

**Fig. 3:**
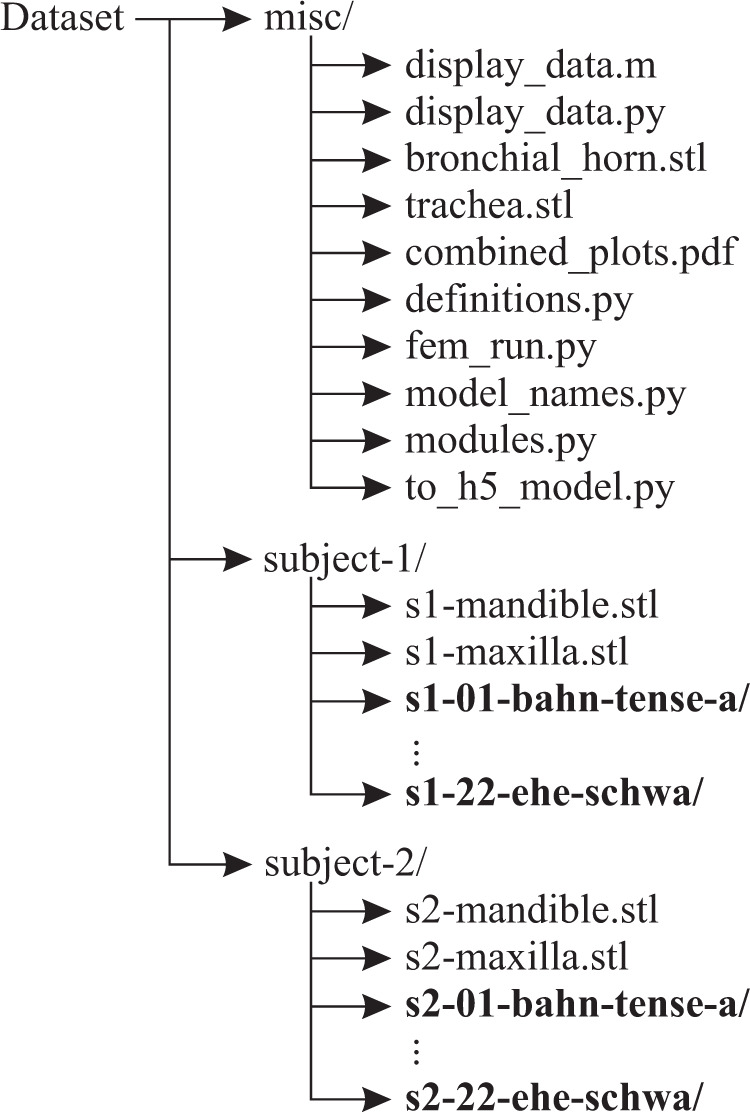
Directory structure of the dataset. Each of the folders written in bold font corresponds to one vocal tract shape and contains the files listed in the Section “Data Records”.

The dataset is available at the platform figshare (10.6084/m9.figshare.c.4869732)^55^ and mirrored at www.vocaltractlab.de/dvtd.zip. The included Matlab and Python scripts are additionally available on GitHub (https://github.com/TUD-STKS/DVTD). The repository has the directory structure shown in Fig. 3, with one directory for the data of subject 1, one for the data of subject 2, and one for miscellaneous. The miscellaneous directory contains the following files:

**display_data.m, display_data.py** are two functionally identical Matlab and Python scripts to display the measured data.

**bronchial_horn.stl, trachea.stl** are 3D-printable models of the subglottal structure that was used for the turbulence noise measurements.

**combined_plots.pdf** contains plots of the VVTFs and the power spectral densities for all models.

**definitions.py, fem_run.py, model_names.py, modules.py, to_h5_model.py** are Python scripts to run the FE simulations (see section “Code Usage”).

The directories for each subject contain two STL files with volume models of the mandible and maxilla, and one sub-directory for each speech sound. Each sub-directory contains the following files (where *XX* is the speech sound label according to Table 1:

***XX*-mri/*ZZ*.ima** are the (raw) MRI files of the vocal tract.

***XX*-inner-surface.stl** represents the inner surface geometry of the vocal tract model in terms of a triangle mesh. The plane of the glottis and the lips are open. The unit of the coordinate values is mm (as for all other STL files).

***XX*-inner-surface-closed.stl** is similar to *XX*-inner-surface.stl, but closed at the glottal end and at the lips. It was the basis for the creation of the finite-element model.

***XX*-fem.msh** contains the 3D finite-element mesh including the definitions of nodes, elements, domain entities and flags for boundary conditions. It is the native file format of the software Gmsh (http://gmsh.info/) to store the numerical models. This file type is supported by a lot of free partial-differential-equation solvers.

***XX*-printable-model.stl** is the complete 3D-printable solid volume model of the vocal tract.

***XX*-printable-left-half.stl** is the left half of the printable volume model.

***XX*-printable-right-half.stl** is the right half of the printable volume model.

***XX*-vvtf-measured.txt** contains the (linear) magnitude and phase (in rad) samples of the measured VVTF (based on the 3D-printed models) from 100 Hz to 10,000 Hz as tabular data. The frequency resolution is 0.961304 Hz. Below 100 Hz and above 10,000 Hz the magnitude and phase samples are set to 1 and 0, respectively.

***XX*-sweep-reference.wav, XX-sweep-primary.wav** contain the reference and primary sweep responses from which the VVTF was obtained. These files can be used with the software MeasureTransferFunction^48^ to retrace the determination of the VVTF.

***XX*-vvtf-calculated.txt** contains the (linear) magnitude and phase (in rad) samples of the calculated VVTF (based on the FE models) from 0 Hz to 10,000 Hz as tabular data. The frequency resolution is 0.961304 Hz (same as for the measured VVTF).

***XX*-noise-500mW.wav, ..., XX-noise-3000mW.wav** Each of these audio files contains a 10 s recording of the noise generated by a stationary flow through the glottis of the model for six values of the fluid power between 500 mW and 3000 mW. The sound was measured 30 cm in front and 30 cm sideways of the mouth. The sampling rate is 48,000 Hz. The samples are encoded as floating point numbers between −1 and +1 and proportional to the sound pressure. The value +1 corresponds to a sound pressure of 12.62 Pa (=116 dB).

***XX*-noise-psd.txt** contains the power spectral densities of the noise measured for the six different fluid power levels as tabular data. The frequency resolution is 46.9 Hz.

***XX*-noise-metadata.txt** contains for each of the six fluid power levels the average volume velocity, the average subglottal pressure, and the sound pressure level of the radiated sound (recorded 30 cm in front and 30 cm sideways from the mouth opening).

***XX*-model-sound.wav** is an audio file that was generated by exciting the 3D-printed vocal tract model with either a vibrating reed source (for the vowels and the lateral) or a constant airflow through the glottis at a subglottal pressure of 800 Pa (for the fricatives). The file was used in the perception experiment for the evaluation. The sampling rate is 48,000 Hz.

***XX*-reference-sound.wav** is an audio recording of the corresponding speech sound by the (real) subject s1 or s2. Due to the high noise level in the MRI scanners, the recordings were performed separately from the MRI scan sessions in a sound-proofed room. The subjects were recorded in a sitting position and were instructed to sustain the speech sounds as similar as possible to the sounds in the scanner. The sampling rate is 44,100 Hz.

The file types used to store the data can be opened with a range of programs on the different operating systems. The following is a list of free and platform-independent software tools (running on Windows, Linux, MacOS) to open the file types:

**IMA files**: itk-SNAP (www.itksnap.org)

**MSH files**: Gmsh (www.gmsh.info)

**STL files**: MeshLab (www.meshlab.net)

**WAV files**: VLC media player (www.videolan.org)

## Technical Validation

### Validation of the methods for transfer function measurements and simulation

**Fig. 4:**
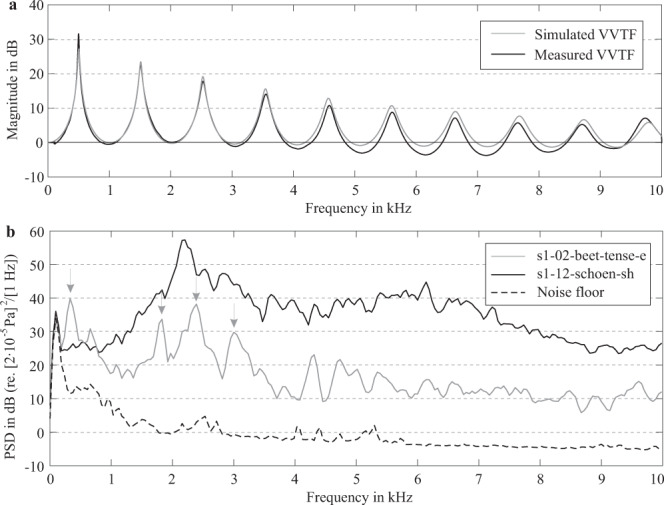
(**a**) Volume velocity transfer functions obtained for a cylindrical tube by measurement (black curve) and finite element simulation (gray curve). (**b**) Power spectral densities of the sound radiated by the models s1-12-schoen-sh (black curve) and s1-02-beet-tense-e (gray curve) generated by a constant flow through the glottis at a fluid power of 500 mW. The gray arrows indicate the first four resonances of the vowel. The dashed line represents the noise floor in the measurement room.

To check the general validity of the transfer function measurements and calculations, we applied them to a cylindrical tube with one open end and compared the obtained resonance frequencies with the corresponding analytical solutions. The tube was designed with a length of $L=165.4$ mm and a radius of $r=8.5$ mm to roughly represent the vowel /ə/. The tube was both 3D-printed and converted into an FEM mesh. Like for all models in the dataset, the VVTF of the printed model was measured and the VVTF of the FE model was calculated. From both transfer functions, the first three resonance frequencies were determined by peak picking. The analytical solution for the resonance frequencies is given by

$${f}_{{\rm{R}}n}=(2n-1)\frac{c}{4(L+\Delta L)},$$

where *n* = 1, 2, 3, ..., *c* = 345 m/s is the sound velocity at 22° C, $L=165.4$ mm is the tube length, and $\Delta L=0.6r=5.1$ mm is the open-end correction of the tube length. The first three resonance frequencies obtained from the analytical calculations, the measurements, and the FEM simulations are summarized in Table 2 and agree very well with a maximal difference of 1.4%. Figure 4a shows the measured and calculated VVTFs. They confirm the high agreement of the resonance frequencies, and furthermore demonstrate the low noise of the measurement (black curve). However, towards high frequencies, differences in magnitude of a few dB occur between the curves. Similar differences exist also for the models of the dataset, and are presumably caused by small differences in the losses or boundary conditions.

**Table 2:** The first three resonance frequencies of the cylindrical tube obtained by the analytical calculation, the FEM simulation, and the measurement using a 3D-printed physical model.

	*f*_R1_	*f*_R2_	*f*_R3_
Calculation (analytic)	506	1518	2529
Calculation (FEM)	503	1511	2523
Measurement	499	1510	2527
Absolute deviation between analytic and FEM results in %	0.6	0.5	0.2
Absolute deviation between analytic and measurement results in %	1.4	0.5	0.1

### Validation of noise measurements

In contrast to the transfer functions, the noise measurements obtained during the aeroacoustic characterization of the vocal tract models (Fig. 2b) could not be compared to corresponding simulation results (such simulations are subject of ongoing research). Instead, we qualified the signal-to-noise ratio (SNR) of the measurements by measuring the noise floor, and validated the absolute scale of the measured audio data to ensure that our custom-made software did not introduce any scaling errors. Two examples of obtained power spectral densities for a vowel and a fricative (both for the lowest fluid power level of 500 mW) are shown in Fig. 4b. Here it can be seen that for frequencies above 200 Hz, the signals are at least 10 dB above the noise floor (dashed line). Furthermore, for the vowel /eː/ of speaker s1 (gray curve) the PSD clearly shows the resonances of the vowel (gray arrows). These peaks correspond well to the resonance frequencies obtained by the VVTF measurements (Table 3).

**Table 3:** Measured (${f}_{{\rm{R}}i{\rm{,m}}}$) and calculated (${f}_{{\rm{R}}i{\rm{,c}}}$) resonance frequencies of the vocal tract models of the male speaker (s1) and their differences in percent.

Sound	*f*_R1,m_ [Hz]	*f*_R1,c_ [Hz]	$\frac{|{\boldsymbol{\Delta }}{{\boldsymbol{f}}}_{{\bf{R1}}}|}{{{\boldsymbol{f}}}_{{\bf{R}}{\bf{1}},{\bf{c}}}}$	${{\boldsymbol{f}}}_{{\bf{R2,m}}}$ [Hz]	${{\boldsymbol{f}}}_{{\bf{R2,c}}}$ [Hz]	$\frac{|{\boldsymbol{\Delta }}{{\boldsymbol{f}}}_{{\bf{R2}}}|}{{{\boldsymbol{f}}}_{{\bf{R2,c}}}}$	${{\boldsymbol{f}}}_{{\bf{R3,m}}}$ [Hz]	${{\boldsymbol{f}}}_{{\bf{R3,c}}}$ [Hz]	$\frac{\left|{\boldsymbol{\Delta }}{{\boldsymbol{f}}}_{{\bf{R3}}}\right|}{{{\boldsymbol{f}}}_{{\bf{R}}{\bf{3}},{\bf{c}}}}$	Average
/aː/	517	514	0.6%	1114	1105	0.8%	2315	2304	0.5%	0.6%
/eː/	253	256	1.2%	1793	1793	0.0%	2360	2313	2.0%	1.1%
/iː/	187	192	2.6%	1803	1794	0.5%	2616	2612	0.2%	1.1%
/oː/	263	261	0.8%	683	677	0.9%	2106	2101	0.2%	0.6%
/uː/	194	197	1.5%	656	661	0.8%	1927	1917	0.5%	0.9%
/εː/	436	435	0.2%	1596	1587	0.6%	2239	2214	1.1%	0.6%
/øː/	236	236	0.0%	1281	1274	0.5%	1809	1801	0.4%	0.3%
/yː/	154	159	3.1%	1324	1327	0.2%	1774	1766	0.5%	1.3%
/l/	238	244	2.5%	1605	1599	0.4%	2273	2267	0.3%	1.0%
/f/	301	306	1.6%	1357	1358	0.1%	2031	2034	0.1%	0.6%
/s/	189	190	0.5%	1325	1322	0.2%	2375	2371	0.2%	0.3%
/∫/	159	150	6.0%	1823	1799	1.3%	2170	2150	0.9%	2.8%
/ç/	152	159	4.4%	1693	1677	1.0%	2636	2599	1.4%	2.3%
/x/	544	532	2.3%	1113	1081	3.0%	2385	2379	0.3%	1.8%
/a/	523	526	0.6%	974	971	0.3%	2382	2374	0.3%	0.4%
/ε/	434	434	0.0%	1537	1526	0.7%	2247	2233	0.6%	0.4%
/I/	296	297	0.3%	1461	1455	0.4%	2055	2050	0.2%	0.3%
/ɔ/	360	360	0.0%	774	769	0.7%	2331	2341	0.4%	0.4%
/ʊ/	252	253	0.4%	661	662	0.2%	2141	2132	0.4%	0.3%
/Y/	303	306	1.0%	1168	1167	0.1%	2052	2050	0.1%	0.4%
/œ/	393	394	0.3%	1215	1208	0.6%	2074	2063	0.5%	0.5%
/ə/	337	329	2.4%	1571	1561	0.6%	2259	2223	1.6%	1.6%
Average:			1.5%			0.6%			0.6%	0.9%

To validate the absolute scale of the measured audio data, the SPLs calculated with Eq. (3) were compared with the readings of a hand-held sound level meter at the position of the measurement microphone. The SPL values agreed within ±2 dB. Accordingly, the audio samples in the noise audio files *XX*-noise-500 mW.wav, …, *XX*-noise-3000 mW.wav closely approximate absolute sound pressure values (the audio sample value 1.0 corresponds to 12.62 Pa).

### Analysis of the resonance frequencies of the vocal tract models

To check the similarity of the calculated and the measured transfer functions for the resonators in the dataset, the first three resonance frequencies ${f}_{{\rm{R}}1}$, ${f}_{{\rm{R}}2}$, and ${f}_{{\rm{R}}3}$ have been extracted from the magnitude spectra. The obtained resonances are given in Tables 3 and 4 for the male and the female speaker, respectively. It can be seen that the frequency deviations are mostly below 1%, with the highest deviation being 6.0% for ${f}_{{\rm{R}}1}$ of the /∫/ of speaker s1. The average absolute deviations across all three resonances and all vowels are 0.9% for s1 and 0.8% for s2. The overall similarity of the simulated and measured transfer functions (also between the resonance frequencies) is also high, as can be visually checked with the provided scripts. As expected, the deviations get stronger with increasing frequency, because local details of the geometry of the vocal tract become more important.

**Table 4:** Measured (${f}_{{\rm{R}}i{\rm{,m}}}$) and calculated (${f}_{{\rm{R}}i{\rm{,c}}}$) resonance frequencies of the vocal tract models of the female speaker (s2) and their differences in percent.

Sound	${{\boldsymbol{f}}}_{{\bf{R1,m}}}$ [Hz]	${{\boldsymbol{f}}}_{{\bf{R1,c}}}$ [Hz]	$\frac{|{\boldsymbol{\Delta }}{{\boldsymbol{f}}}_{{\bf{R1}}}|}{{{\boldsymbol{f}}}_{{\bf{R}}{\bf{1}},{\bf{c}}}}$	${{\boldsymbol{f}}}_{{\bf{R2,m}}}$ [Hz]	${{\boldsymbol{f}}}_{{\bf{R2,c}}}$ [Hz]	$\frac{|{\boldsymbol{\Delta }}{{\boldsymbol{f}}}_{{\bf{R2}}}|}{{{\boldsymbol{f}}}_{{\bf{R}}{\bf{2}},{\bf{c}}}}$	${{\boldsymbol{f}}}_{{\bf{R3,m}}}$ [Hz]	${{\boldsymbol{f}}}_{{\bf{R3,c}}}$ [Hz]	$\frac{|{\boldsymbol{\Delta }}{{\boldsymbol{f}}}_{{\bf{R3}}}|}{{{\boldsymbol{f}}}_{{\bf{R}}{\bf{3}},{\bf{c}}}}$	Average
/aː/	752	758	0.8%	1414	1393	1.5%	3153	3149	0.1%	0.8%
/eː/	319	326	2.1%	2487	2481	0.2%	2972	2925	1.6%	1.3%
/iː/	217	225	3.6%	2502	2501	0.0%	3506	3515	0.3%	1.3%
/oː/	415	419	1.0%	902	902	0.0%	3171	3167	0.1%	0.4%
/uː/	335	334	0.3%	870	857	1.5%	2729	2728	0.0%	0.6%
/εː/	680	678	0.3%	1900	1899	0.1%	2845	2825	0.7%	0.4%
/øː/	387	388	0.3%	1600	1593	0.4%	2324	2317	0.3%	0.3%
/yː/	300	303	1.0%	1711	1699	0.7%	2263	2265	0.1%	0.6%
/l/	304	318	4.4%	1773	1772	0.1%	2870	2859	0.4%	1.6%
/f/	484	488	0.8%	1613	1613	0.0%	2561	2559	0.1%	0.3%
/s/	297	304	2.3%	1652	1646	0.4%	2979	2970	0.3%	1.0%
/∫/	299	308	2.9%	1781	1785	0.2%	2994	2994	0.0%	1.0%
/ç/	326	333	2.1%	2371	2371	0.0%	3128	3122	0.2%	0.8%
/x/	731	733	0.3%	1545	1517	1.8%	2860	2843	0.6%	0.9%
/a/	693	688	0.7%	1208	1189	1.6%	2998	2977	0.7%	1.0%
/ε/	578	573	0.9%	1718	1695	1.4%	2704	2675	1.1%	1.1%
/I/	363	368	1.4%	1980	1966	0.7%	2633	2567	2.6%	1.5%
/ɔ/	468	470	0.4%	1075	1063	1.1%	3079	3071	0.3%	0.6%
/ʊ/	379	386	1.8%	1093	1079	1.3%	3004	2982	0.7%	1.3%
/Y/	415	417	0.5%	1722	1712	0.6%	2452	2455	0.1%	0.4%
/œ/	534	532	0.4%	1477	1453	1.7%	2423	2404	0.8%	0.9%
/ə/	510	512	0.4%	1503	1500	0.2%	2576	2574	0.1%	0.2%
Average:			1.3%			0.7%			0.5%	0.8%

**Fig. 5:**
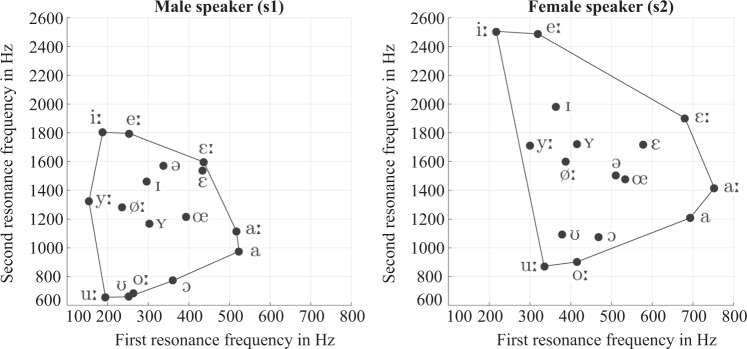
Measured resonance frequencies of the vowels.

The plots in Fig. 5 show the first two measured resonance frequencies of the vowels. They show that the vowels are arranged in the typical way known from formant plots^45^, especially along the periphery of the vowel space (although resonances and formants are not identical concepts and their frequencies may differ^56^). The relative order of the resonance frequencies of the lax vowels partly differs between the two speakers, for example, ${f}_{{\rm{R}}2}$ of /ə/ is comparatively high for s1, and more in the center of the vowel space for s2. However, the plots confirm that all vowels substantially differ from each other, i.e, no two vowels were produced in exactly the same way. Hence, the recorded samples provide a good coverage of the vowel space.

### Perceptual evaluation

#### Method

To ascertain the perceptual validity of the vocal tract models, a perception experiment was conducted where listeners were asked to identify the phonemes from the sounds that were synthesized with the 3D-printed vocal tract models (files *XX*-model-sound.wav). As a baseline, also the natural productions of the phonemes were included in the experiment (files *XX*-reference-sound.wav). All speech sounds were separated into three groups that were used in different tasks of the experiments: the tense vowels /aː, eː, iː, oː, uː, εː, øː, yː/, the lax vowels /a, ε, I, ɔ, ʊ, Y, œ/, and the voiceless fricatives /f, s, ∫, ç, x/.

Twenty native German speakers (11 male, 9 female, 20–42 years, median age 25.5 years) participated in the perception experiment. All subjects gave informed consent and none of them reported hearing or speaking problems. Each subject conducted the experiment individually at a computer in a soundproofed room. The experiment consisted of three tasks that were designed with the software Praat^57^ and performed in sequence.

The first task was the identification of the tense vowels. Here, all 32 stimuli of the tense vowels (2 subjects × {natural vs. synthetic} × 8 vowels) were successively played to the participant in an individual randomized order five times for a total of 160 presentations. After each presentation, the participant had to select the perceived vowel by clicking one of eight buttons on the computer screen (forced choice). Each button was labeled with a German word where the target vowel was emphasized (e.g., the word “b AH n” for the vowel /aː/). Each stimulus could be repeated up to five times by subject request, and there was a “back” button to allow corrections in the case of “mis-clicks”. The stimuli were played using a USB audio interface (Aureon XFire 8.0 HD, volume setting 60/100) over semi-open headphones (AKG K240 Monitor).

The second task was the identification of the lax vowels. Here, all 28 stimuli of the lax vowels (2 subjects × {natural vs. synthetic} × 7 vowels) were successively played to the participant in an individual randomized order five times for a total of 140 presentations. As before, the participant had to select the perceived vowel by clicking the accordingly labeled button after each presentation with the same options to go back or repeat.

The third task was the identification of the voiceless fricatives analogous to the tasks 1 and 2. This task contained 20 different stimuli (2 subjects × {natural vs. synthetic} × 5 fricatives) that were presented five times each for a total of 100 presentations. The total experiment took about 30 min.

## Results

**Fig. 6:**
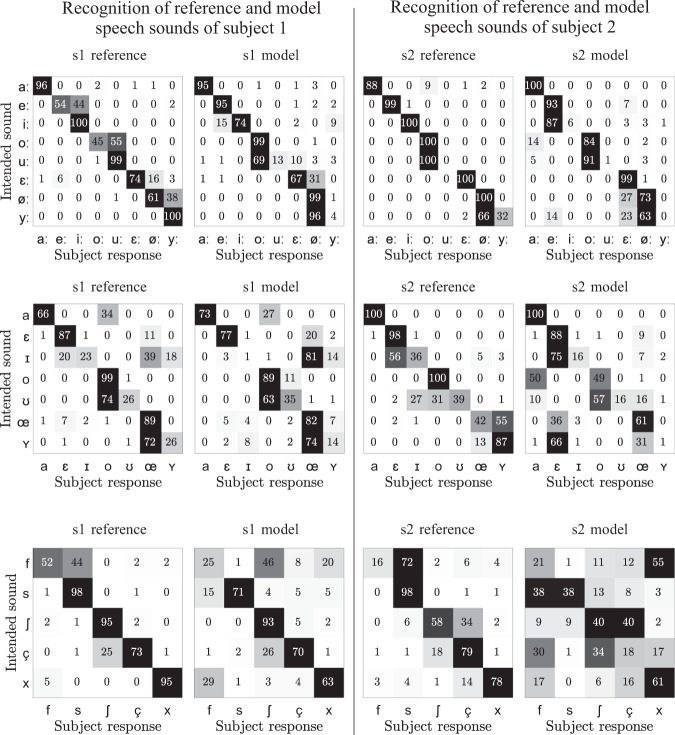
Confusion matrices obtained from the listening experiment with the naturally-produced stimuli (reference) and the artificially generated stimuli (model) using the 3D-printed vocal tract models. The left two columns of subpanels show the confusion matrices for subject 1, and the right two columns for subject 2. The top, middle, and bottom rows of subpanels show the results for the tense vowels, the lax vowels, and the fricatives, respectively. As each stimulus was rated five times by 20 listeners, the numbers in the matrices are both absolute values and percentage values.

The results of the three forced-choice tasks are presented in terms of confusion matrices in Fig. 6. As each stimulus was rated five times by 20 listeners, the numbers in the matrices are both absolute values and percentage values. For subject 1, the recognition rate was 68.3% (78.6%) for the model (reference) stimuli of the tense vowels, 53.0% (60.7%) for the model (reference) stimuli of the lax vowels, and 64.4% (82.6%) for the model (reference) stimuli of the fricatives. For subject 2, the recognition rate was 57.0% (77.4%) for the model (reference) stimuli of the tense vowels, 47.3% (71.7%) for the model (reference) stimuli of the lax vowels, and 35.6% (65.8%) for the model (reference) stimuli of the fricatives. Hence, for both subjects and all three groups of stimuli, the synthesized stimuli were recognized worse than the natural stimuli. However, Fig. 6 shows that the patterns of confusion of the speech sounds were quite similar between the natural and synthetic stimuli.

The lower recognition rate of the synthesized phonemes may have multiple reasons:

- The vocal tract models were 3D-printed with hard plastic walls. In reality, the vocal tract walls are softer and lead to a stronger damping of the resonances.
- The nasal port was closed in all vocal tract models. In reality, vowels are often produced with a slightly open nasal port (especially the lower vowels), which affects the overall spectral shape and hence the perception.
- All synthetic vowels were excited with the same vibrating reed source, which was tuned to a typically male fundamental frequency. The low-frequency excitation of the (smaller) female vocal tract models (speaker 2) may have led to perceptual confusions, as this combination occurs rarely in natural speech.
- For the fricatives, the cross-sectional area of the critical constriction is very important for a realistic noise spectrum. The low recognition rates of some model sounds (especially /f/) may be due to overly smoothed vocal tract geometries around the constrictions which distorted the noise spectra.

Despite these limitations, many vowels and fricatives were successfully synthesized, indicating valid and representative vocal tract shapes of German speech sounds in the dataset.

## Usage Notes

### Running the Finite-Element Code

This section describes how to run the FE simulation on the basis of the files *XX*-fem.msh to generate the VVTF files *XX*-vvtf-calculated.txt. Possible reasons to re-run the simulations may be the need to evaluate the sound pressures or volume velocities at different positions in the FEM mesh, to generate the spectra with a different frequency resolution, or to extend the equations of the simulation. Running the simulations requires the software FEniCS (https://fenicsproject.org/), which you must install prior to running the scripts using the guide appropriate for your OS from the website. The subsequent steps were tested under Ubuntu versions 16.04 and 18.04. The first step is the conversion of the MSH file of a model into an XML file by typing

### **dolfin-convert XX-fem.msh XX.xml**

on the command line (where XX is the sound label, as before). Make sure to omit “-fem” from the XML file name. The XML must otherwise have the identical name. Using the provided python script to_h5_model.py (in the misc subdirectory), this XML file must be converted into a H5 file, which allows the parallel execution of the simulation:

### **python3 to_h5_model.py XX(filename without extension)**

Both, the XML and the H5 version of the model contain all necessary information about the surface-IDs and the volume-IDs, which are needed for the FE simulation. To calculate the VVTF for a model, the provided Python script fem_run.py must be called:

### **python3 fem_run.py**

The specific subject and model to simulate must be specified in the file definitions.py. The simulated VVTF is written to the file *XX*-vvtf-calculated.txt in the respective subfolder.

## Data Availability

All the software used in this study are open source (see the previous sections for references or URLs). In addition, the dataset contains several custom-made Matlab and Python scripts (see section “Data Records”).
